# Cardiff cardiac ablation patient-reported outcome measure (C-CAP): validation of a new questionnaire set for patients undergoing catheter ablation for cardiac arrhythmias in the UK

**DOI:** 10.1007/s11136-015-1194-1

**Published:** 2015-12-10

**Authors:** Judith White, Kathleen L. Withers, Mauro Lencioni, Grace Carolan-Rees, Antony R. Wilkes, Kathryn A. Wood, Hannah Patrick, David Cunningham, Michael Griffith

**Affiliations:** Cedar, Cardiff University, Cardiff Medicentre, Heath Park, Cardiff, CF14 4UJ UK; Cedar, Cardiff & Vale University Health Board, Cardiff Medicentre, Heath Park, Cardiff, CF14 4UJ UK; University Hospitals Birmingham NHS Foundation Trust, Birmingham, UK; Duke University School of Nursing, 307 Trent Drive, DUMC 3322, Durham, NC 27710 USA; National Institute for Health and Care Excellence, 10 Spring Gardens, London, SW1A 2BU UK; National Institute for Cardiovascular Outcomes Research (NICOR), UCL Institute of Cardiovascular Science, 3rd Floor 170 Tottenham Court Road, London, W1T 7HA UK

**Keywords:** Cardiac ablation, Quality of life (QoL), Patient-reported outcome measures (PROMs), Arrhythmia, Validation, Questionnaires

## Abstract

**Purpose:**

To formally test and validate a patient-reported outcome measure (PROM) for patients with cardiac arrhythmias undergoing catheter ablation procedures in the UK [Cardiff Cardiac Ablation PROM (C-CAP)].

**Methods:**

A multicentre, prospective, observational cohort study with consecutive patient enrolment from three UK sites was conducted. Patients were sent C-CAP questionnaires before and after an ablation procedure. Pre-ablation C-CAP1 (17 items) comprised four domains: patient expectations; condition and symptoms; restricted activity and healthcare visits; medication and general health. Post-ablation C-CAP2 (19 items) comprised five domains including change in symptoms and procedural complications. Both questionnaires also included the generic EQ-5D-5L tool (EuroQol). Reliability, validity, and responsiveness measures were calculated.

**Results:**

A total of 517 valid pre-ablation and 434 post-ablation responses were received; questionnaires showed good feasibility and item acceptability. Internal consistency was good (Cronbach’s alpha >0.7) and test–retest reliability was acceptable for all scales. C-CAP scales showed high responsiveness (effect size >0.8). Patients improved significantly (*p* < 0.001) following ablation across all disease-specific and global scales. Minimal clinically important difference was calculated. Improvement beyond the smallest detectable change of 9 points (symptom severity scale), 3 points (frequency and duration of symptoms scale), and 8 points (impact on life scale) indicates an important change. Amendments to the C-CAP questionnaires were identified through the validation process and made to produce the final tools.

**Conclusions:**

The final C-CAP questionnaires are valid, reliable, and responsive tools for measuring symptom change, impact, and expectations in patients undergoing ablation for cardiac arrhythmias. C-CAP questionnaires provide a tool with disease-specific and generic domains to explore how cardiac ablation procedures in the UK impact upon patients’ lives.

**Electronic supplementary material:**

The online version of this article (doi:10.1007/s11136-015-1194-1) contains supplementary material, which is available to authorized users.

## Introduction

Cardiac arrhythmias are experienced by more than 1 million people a year in the UK. Arrhythmia-related symptoms include palpitations, breathlessness, chest pain, dizziness, and fatigue [1], which can have profoundly negative effects on patients’ quality of life (QoL) [2]. The cost to the UK National Health Service (NHS) of treating people with atrial fibrillation (AF), the most common of these arrhythmias, is large; estimates put the direct cost at 0.9–2.4 % of overall healthcare expenditure [3]. The intended benefit of percutaneous radiofrequency cardiac ablation is to improve patient QoL and eliminate or reduce arrhythmia-related symptoms.

Patient-reported outcome measures (PROMs) aim to determine a patient’s own view of their symptoms, functional status, and health-related QoL before and after an intervention. PROMs offer a particularly useful platform to evaluate the effect of cardiac ablation on arrhythmia-related symptoms because arguably the biggest impact of a successful ablation treatment is an alleviation of anxiety and physical symptoms [4].

Patient-reported outcome measures (PROMs) should include both a generic and a disease-specific tool; both can be used simultaneously to build a comprehensive picture of patients’ status [5]. Whilst generic QoL instruments such as SF-36 and EQ-5D-5L have been validated and extensively used in a variety of populations, formal validation of disease-specific tools is not always often performed. Many QoL questionnaires have been developed specifically for use in patients with AF [4, 6]. The questionnaires validated in this study provide a method of including both generic and disease-specific measures combined with measures of patient expectations and experiences of their ablation procedure into one questionnaire.

The key elements of validation involve evaluating a PROM tool for its reliability (test–retest, internal consistency), validity (content and construct), sensitivity (to differences between groups), and responsiveness (to change in patients’ condition) [7]. These steps are essential to enable data derived from a tool to be useful and interpretable. The aim of this study is to formally validate a PROM tool for UK patients with arrhythmias treated with catheter ablation. This builds on a previous feasibility study [1] and the initial stage of this study which was to establish content validity through patients’ interviews [8].

## Methods

This multicentre, prospective, observational cohort study was designed to formally develop, evaluate, and validate a new UK PROM tool for patients with cardiac arrhythmias treated with cardiac ablation procedures (UK Clinical Research Network Study Portfolio reference 13148).

### Ethics

The study protocol was reviewed and approved by the Nottingham 1 Research Ethics Proportionate Review Sub-Committee (reference 12/EM/0164) and conducted in accordance with Good Clinical Practice and the Declaration of Helsinki. Informed consent was obtained from all patients who took part in this study.

### Development of the initial C-CAP questionnaires

Initial item generation, face validity, and content validity of the cardiac ablation PROM were evaluated as described previously [1, 8]. The pre-validation C-CAP questionnaires used in this study consist of a 17-item pre-ablation questionnaire (C-CAP1) and a 19-item post-ablation questionnaire (C-CAP2) and are described in full by Withers et al. [8]. Questionnaires which incorporate the amendments identified in the current validation study are termed “final C-CAP1 and final C-CAP2” for clarity and have been provided as an online resource.

### Patients

Patients under the care of physicians at three clinical sites in the UK (University Hospital Wales, Cardiff; Queen Elizabeth Hospital, Birmingham; Freeman Hospital, Newcastle-Upon-Tyne) were eligible for inclusion in this study. Patients were enrolled only if they were aged 18 or over, had a diagnosis of symptomatic cardiac arrhythmia, had consented to and were awaiting a cardiac ablation procedure, and were able to read, write, and understand English or Welsh.

### Questionnaire procedures

Patients across three sites were approached consecutively to take part in the study and provided with a participant information sheet, consent form, the pre-validation, pre-ablation questionnaire (C-CAP1), and a stamped addressed envelope at the time of their appointment or with their appointment letter. Patients were given time to consider their involvement and completed the C-CAP1 questionnaire if and when they wished to do so (some completed the questionnaire on the day of their ablation). Patients from whom a signed consent form was received were considered to be enrolled. Returned C-CAP1 questionnaires were excluded from analysis if the patient had received their ablation procedure prior to completion of the questionnaire.

No change in treatment or clinical assessment was carried out on patients as a result of their participation in this study. Patients underwent percutaneous radiofrequency or cryotherapy ablation using conscious sedation or a general anaesthetic.

At 8–16 weeks following their ablation, patients were sent a pre-validation, post-ablation questionnaire (C-CAP2) to their home with a freepost envelope. Non-responders were sent a reminder letter with a replacement questionnaire approximately 2–3 weeks after the initial mailing. C-CAP2 questionnaires were excluded from analysis if they were completed more than 20 weeks after the ablation.

Identical retest questionnaires (C-CAP1R and C-CAP2R) were sent to a random subset of patients (no patients were sent retest questionnaires for both). Pre-ablation retest questionnaires (C-CAP1R) were sent 1 week after completion of C-CAP1. The following exclusions were applied to select for patients who were assumed to have a stable condition between completing test and retest questionnaires: (1) an ablation was carried out in between completion of C-CAP1 and C-CAP1R; (2) >30 days elapsed between the patient completing C-CAP1 and C-CAP1R. Post-ablation retest questionnaires (C-CAP2R) were sent to patients 1 week after completion of C-CAP2. Returned C-CAP2R questionnaires were excluded if >30 days passed between completion of C-CAP2 and C-CAP2R.

Further follow-up is currently being conducted at 1 and 5 years post-procedure (data not included in this publication).

### Sample size

No formal sample size calculation was conducted for this questionnaire validation study. A minimum sample size of 150 patients from each centre has been suggested in previous studies to allow meaningful comparisons to be made [9]. A target of 450 enrolled patients was set to ensure that smaller sub-groups are adequately represented within the sample and to provide representation from various arrhythmia types.

### Description of C-CAP questionnaires

Pre-validation C-CAP1 was split into four domains (Table 1) and comprised 17 questions related to patient expectations; condition and symptoms; activity and healthcare visits; and medication and general health. The conditions and symptoms domain contained three multi-item scales: (1) symptom severity (15 sub-items); (2) frequency and duration of symptoms (2 sub-items); (3) impact on life (10 sub-items). Pre-validation C-CAP2 comprised five domains (Table 1), three of which are replicated from C-CAP1 and allowed comparison before and after the procedure (condition and symptoms; activity and healthcare visits; and medication and general health). In addition C-CAP2 covered change in symptoms and procedure-related symptoms. Both questionnaires also include the generic EuroqoL EQ-5D-5L [10] questionnaire and visual analogue scale (EQ-VAS). The EQ-5D-5L, used since the beginning of this project, was chosen over the EQ-5D-3L (used in other NHS PROM tools) because of its improved discriminatory power which we felt was important during development and testing of these questionnaires [11].

**Table 1:** Description of domains within the pre-validation C-CAP questionnaires used in this study

Domain	Question/item numbers	Domain description
Pre-validation, pre-ablation questionnaire (C-CAP1)		
Pre-ablation patient expectations	1–5	Contains a 4 item Likert scale (Q1–3b) with five response options (each item scored 0–4); each explored patients’ treatment expectations prior to the procedure. The “treatment expectations” multi-item scale had a minimum score of 0 and a maximum of 16 (the 4 items in the scale were given equal weight, and each had a minimum score of 0 and a maximum of 4). This domain also asked whether this is the patient’s first ablation (Q4) and for the number of previous ablations received (Q5)
Condition and symptoms	6, 7, 8, 13	This domain was a modified version of the disease-specific Patient Perception of Arrhythmia Questionnaire (PPAQ) originally developed by Wood et al. [21]. Following adaptations for use in a UK population with specialist, lay and patient input, this updated tool included elements which were divided into three multi-item scales where a high score reflects a worse health state: Symptom severity (Q6a–o): 15 item symptom scale, each symptom/item had 4 response options (scored 0–3). The minimum score was 0 and the maximum was 45 (equal weight was given to each item in the scale and all subsequent scales) Frequency and duration of symptoms (Q7–8): two item scale, each item had 5 response options (scored 0–4). The minimum score was 0 and the maximum was 8 Impact on life (Q13a–j): 10 item scale, each item had 4 response options (scored 0–3). The minimum score was 0 and the maximum score was 30
Restricted activity days and healthcare visits	9a–12b	This domain was modified from the PPAQ [20] and aimed to count how many days (either work/school/college, social activities, or normal daily activities) in the last 30 have been affected by arrhythmia symptoms. The number of visits to a GP or hospital in the last 30 days was also recorded
Medication and general health	14–17	Q14 asked whether the respondent normally takes medication (yes/no); Q15 asked for the name and dose of medication (free text); Q16 asked how important a reduction in medication is for the respondent (Likert scale with 4 response categories scored from 0 to 3); Q17 asked whether the respondent had been diagnosed with any one of a list of 12 common conditions (with a “tick all that apply” instruction)
EQ-5D-5L	Not numbered	This comprised the widely used global health questionnaire which provides a simple descriptive profile and a single index value for health status [10]. The EQ-5D-5L questionnaire consists of five questions related to mobility, self-care, usual activities, pain/discomfort, and anxiety/depression. Each question can be answered on five different levels. The EQ-5D-5L also includes a visual analogue scale (question 19) from 0 (worst health imaginable) to 100 (best health imaginable). Therefore, a higher score is related to a better outcome, in contrast to the other scoring systems used elsewhere in this paper
Pre-validation post-ablation questionnaire (C-CAP2)		
Post-ablation change in symptoms	1–3b, 7	This domain consists of 4 items each with 4 available responses relating to changes in patients’ arrhythmia-related symptoms since receiving a procedure to treat their condition (scored from 1 to 4 for each scale). Therefore, the change in symptoms multi-item scale has a minimum score of 0 and a maximum of 16. This domain also asks whether the outcome of the procedure met the patients’ expectations
Procedure-related complications	4–6	This domain comprised a binary question relating to whether patients experienced any ablation-related complications and two tables asking patients whether they were warned of or experienced any of a list of complications
Condition and symptoms	8, 9, 10, 15	As described for C-CAP1
Restricted activity days and healthcare visits	11a–13b	As described for C-CAP1
Medication and general health	16–19	As described for C-CAP1
EQ-5D-5L	Not numbered	As described for C-CAP1

C-CAP, Cardiff Cardiac Ablation PROM (patient-reported outcome measure); PPAQ, Patient Perception of Arrhythmia Questionnaire

### Data management and statistics

Patients completed the C-CAP questionnaires by hand in their own time, and responses were entered by researchers at Cedar Healthcare Technology Research Centre within the UK NHS (Cardiff and Vale University Health Board) into the National Audit of Cardiac Rhythm Management (NACRM) database administered by National Institute for Cardiovascular Outcomes Research (NICOR) at University College London. All data entered onto the database were checked for accuracy by a second researcher. Missing data were not imputed. Data were exported from NACRM and were analysed using IBM SPSS Statistics version 21. All statistical tests were two-sided, and p values less than 0.05 were considered statistically significant.

### Validation of C-CAP instrument

#### Feasibility and acceptability

Feasibility was assessed as the proportion of patients who, following enrolment, returned questionnaires within the required timeframe. Acceptability of individual items and multi-item scales was assessed by patient response rate and missing data. Ceiling and floor effects were evaluated as the proportion of patients who responded with the minimum and maximum scores for each dimension.

#### Reliability

Cronbach’s alpha was used to assess internal consistency for disease-specific multi-item scales in C-CAP1 and C-CAP2 (those with ≤2 items were excluded). Coefficients above 0.7 were acceptable, 0.8 (good), and 0.9 (excellent) [12].

Intraclass correlation coefficient (ICC) was used to evaluate test–retest reliability. Scales with an ICC of ≥0.7 were considered to have good reliability. Bland–Altman plots [13] were produced for multi-item scales. For binary items, repeatability was assessed using the kappa coefficient (*κ*) [7].

#### Validity

Content validity was evaluated using one-to-one cognitive interviewing of patients as described by Withers et al. [8]. Convergent validity was tested by comparing the multi-item scales in the condition and symptoms domain (symptom severity; frequency and duration of symptoms; impact on life) to validated global health scores (EQ-5D-5L index and EQ-VAS scale). Correlation coefficients of 0.4–0.7 [Spearman’s Rho (*ρ*)] are considered moderate. We expected that correlations between disease-specific multi-item scales within C-CAP questionnaires would be higher than the correlation between C-CAP scales and global health scores. Discriminant validity was tested by comparing scales in C-CAP questionnaires relating to symptoms and impact against a multi-item scale relating to patient expectations of the results of the procedure. It was assumed that these domains measure different concepts and therefore low correlations (<0.4) were expected.

### Responsiveness and minimal clinically important difference (MCID)

Several distribution-based methods [effect size (ES), standardised response mean (SRM), relative efficiency (RE)] were used to evaluate changes in C-CAP scores following ablation. For both ES and SRM, values of 0.20, 0.50, and 0.80 represent the limits of small, moderate, and large change, respectively [14].

Standard error of measurement (SEM) is a measure of the precision of the instrument. The smallest detectable change (SDC) was calculated from the SEM; it reflects the smallest within-person change in score (*p* < 0.05) that can be interpreted as a real change above measurement error [15] SDC *=* 1.96*√2*SEM.

Minimal clinically important difference (MCID) is defined as the smallest difference in score in the domain of interest which patients perceive as beneficial [16]. Four anchor questions were used to estimate MCID in the case of C-CAP: patients who reported that their symptoms became less frequent; those who reported that the duration of their arrhythmia episodes became shorter; patients whose expectations were met; or patients who reported a global health score improvement of 20 points were considered appropriate to show minimal important change.

## Results

### Questionnaire feasibility

**Fig. 1:**
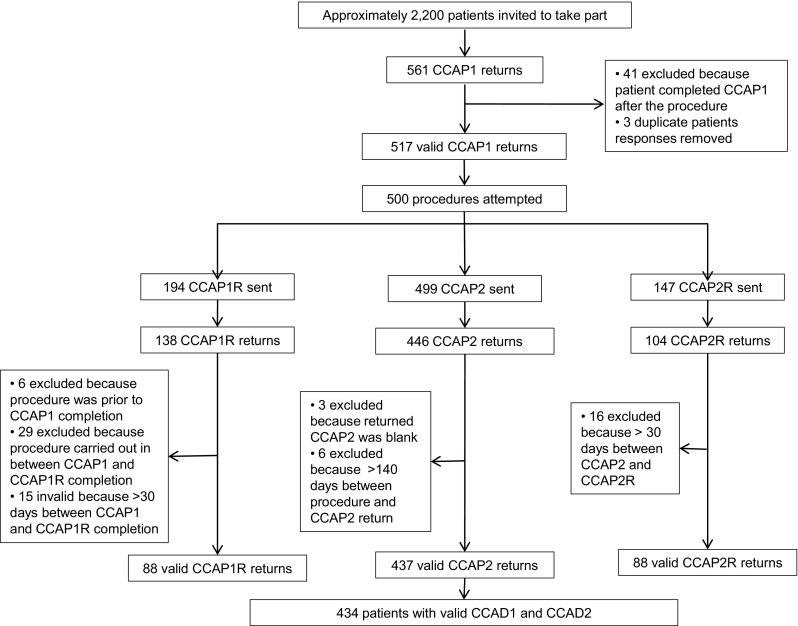
Patient response numbers to Cardiff Cardiac Ablation PROM (C-CAP) questionnaires

Between March 2013 and August 2014 approximately 2200 eligible patients were invited to take part in the study (estimated based on the number of questionnaire packs supplied to clinical teams). Of these, 561 completed pre-validation C-CAP1 questionnaires, of which 517 were valid (Fig. 1). Respondents were 56 % male, with a mean age of 60 years [standard deviation (SD) 13]. The majority of participants were treated for AF (47 %), and 22 % of patients had undergone previous catheter ablations (Table 2). A total of 437 valid pre-validation C-CAP2 (post-ablation) questionnaires were returned (Fig. 1) and 434 patients returned both valid C-CAP1 and C-CAP2 questionnaires. There was a mean of 49 days [SD 52; median 36; interquartile range (IQR) 7–82] between completion of the C-CAP1 questionnaire and the ablation procedure, and then a mean of 77 days (SD 16; median 72; IQR 65–83) between the procedure and completion of C-CAP2. We did not compare patients who were approached by clinicians to those who were eventually enrolled. Consent was given once patients returned their C-CAP1 questionnaire, and therefore, we would not be able to analyse the records of patients who did not return a questionnaire.

**Table 2:** Demographics of enrolled patients and those with valid C-CAP1 and C-CAP2 questionnaires

Demographic	Enrolled patients (*n* = 517)	Patients with valid C-CAP1 and C-CAP2 (*n* = 434)
Male/female	288/227 (56 %/44 %)	246/187 (57 %/43 %)
Mean/median age (years)	60 (SD 13)/62 (IQR 52–68)	61 (SD 12)/63 (IQR 54–68)
Arrhythmia substrate		
Atrial fibrillation	245 (47 %)	214 (49 %)
AVNRT	75 (15 %)	70 (16 %)
Atrial flutter (all)	79 (15 %)	72 (17 %)
Uncommon	10	9
Accessory pathways (all)	32 (6 %)	27 (6 %)
Overt	16	12
Concealed	8	7
Other	8	8
Ventricular extrasystoles/ectopics	12 (2 %)	8 (2 %)
Ventricular tachycardia	12 (2 %)	11 (3 %)
Missing	62 (12 %)	32 (7 %)
Previous intervention		
None	188 (36 %)	162 (37 %)
Percutaneous ablation	113 (22 %)	99 (23 %)
Pacemaker fitted	22 (4 %)	16 (4 %)
Coronary angioplasty	6 (1 %)	5 (1 %)
Cardiac surgery	14 (3 %)	13 (3 %)
Other	1 (0 %)	1 (0 %)
Missing	173 (33 %)	138 (32 %)

AVNRT, atrioventricular nodal re-entry tachycardia

### Item acceptability

The pre- and post-ablation symptom severity scale had a response rate of 83.8 % (433 patients of 517) and 83.3 % (364 of 437 patients), respectively. The pre- and post-ablation impact on life scale had a response rate of 93.4 % (483 patients of 517) and 90.4 % (395 patients of 437), respectively (Table 3). Individual items within these C-CAP1 and C-CAP2 multi-item scales had at least 90 % response rate.

**Table 3:** Acceptability measures for multi-item and single-item questions within C-CAP1 and C-CAP2

	Score mean	Missing data	Floor/ceiling effects^a^
C-CAP1 (pre-ablation) *n* = 517			
Q1–3b: treatment expectations (4 items; score range 0–16)	4.9 (SD 2.0)	3.5 %	0.6 %/0.2 %
Q4: first ablation procedure (1 item; binary)	71 % (Y); 27 % (N)	1.4 %	N/A
Q6: symptom severity index (15 items; score range 0–45)	15.6 (SD 8.6)	16.2 %	1.8 %/0.0 %
Q7–8: frequency and Duration (2 items; score range 0–8)	4.6 (SD 2.0)	3.1 %	7.4 %/9.2 %
Q9: days impact (work/school) (1 item; score range 0–30)	6.1 (SD 10.3)	2.1 % (65.0 % N/A^b^)	52.9 %/12.9 %
Q10: days impact (social activities) (1 item; score range 0–30)	8.6 (SD 10.3)	3.9 % (34.4 % N/A^b^)	27.0 %/12.2 %
Q11: days impact (normal activities) (1 item; score range 0–30)	8.7 (SD 10.4)	4.1 % (30.2 % N/A^b^)	26.0 %/13.5 %
Q12a: GP visits in last 30 days (1 item; score range 0–30)	0.6 (SD 1.1)	25.0 %	66.0 %/0 %
Q12b: hospital visits in last 30 days (1 item; score range 0–30)	0.7 (SD 1.0)	22.8 %	58.4 %/0 %
Q13: impact on life (10 items; score range 0–30)	13.8 (SD 7.5)	6.6 %	1.4 %/0.3 %
Q14: medication taken for arrhythmia (1 item; binary)	82 % (Y); 18 % (N)	0.4 %	N/A
Q16: importance of reducing medication (1 item; score range 0–3)	N/A = 1 % Not important = 7 %; Quite important = 24 %; Very important = 48 %	19.9 %	N/A
EQ-5D-5L (5 items; score range −0.594 to 1.000)	0.7 (SD 0.2)	2.5 %	0.0 %/15.5 %
EQ-VAS (1 item; score range 0–100)	65.2 (SD 18.9)	0.8 %	0.0 %/1.2 %
C-CAP2 (post-ablation) *n* = 437			
Q1–3b: symptom change (4 items; score range 4–16)	8.0 (SD 3.3 %)	5.5 %	15.3 %/2.2 %
Q4: ablation-related complications (1 item; binary)	24 % (Y); 70 % (N)	6.2 %	N/A
Q7: expectations met or exceeded (1 item; binary)	68 % (Y); 26 % (N)	6.2 %	N/A
Q8: symptom severity index (15 items; score range 0–45)	8.8 (SD 7.8)	16.7 %	8.8 %/0.0 %
Q9–10: frequency and duration (2 items; score range 0–8)	2.6 (SD 2.6)	3.2 %	42.6 %/6.4 %
Q11: days impact (work/school) (1 item; score range 0–30)	5.4 (SD 10.6)	2.7 % (68.4 % N/A^b^)	69.8 %/12.7 %
Q12: days impact (social activities) (1 item; score range 0–30)	5.7 (SD 9.6)	4.3 % (47.6 % N/A^b^)	52.9 %/9.5 %
Q13: days impact (normal activities) (1 item; score range 0–30)	5.2 (SD 9.1)	5.3 % (45.3 % N/A^b^)	53.9 %/8.3 %
Q14a: GP visits in last 30 days (1 item)	0.4 (SD 0.9)	14.6 %	75.1 %/0 %
Q14b: hospital visits in last 30 days (1 item)	0.3 (SD 1.1)	22.9 %	81.6 %/0 %
Q15: impact on life (score range 0–30)	7.2 (SD 7.5)	9.6 %	14.9 %/0.3 %
Q16: medication taken for arrhythmia (1 item; binary)	63 % (Y); 35 % (N)	1.6 %	N/A
Q18: medication intake compared with before procedure (1 item; 3 response options)	More = 11 %; same = 35 %; less = 17 %	36.4 %	N/A
EQ-5D-5L (score range −0.594 to 1.000)	0.8 (SD 0.2)	2.5 %	0.0 %/30.5 %
EQ-VAS (score range 0–100)	72.6 (SD 20.1)	1.1 %	0.0 %/3.9 %

^a^Lowest/highest scale scores (of respondents)

^b^Percentage of patients who recorded a response of N/A

High patient numbers reported “not applicable” to questions relating to number of days that symptoms have impacted upon activities (Table 3). We considered N/A a valid response for “days you have missed at work/school/college” (i.e. for patients who are retired or unemployed), but not for questions relating to “social activities” and “normal daily activities”.

There was a high proportion of missing data for questions relating to medication expectations (Table 3) which was almost entirely accounted for by patients responding to an earlier question that they do not normally take medication for their palpitations.

The free text questions relating to medication name and dosage presented difficulties in interpretation. There was variation in how patients described both the name (i.e. generic, brand, descriptive) and dose (strength, number of tablets) which made consistent data entry less reliable.

### Ceiling and floor effects

Ceiling effects of >15 % of respondents reporting maximum scores in multi-items scales were observed in both pre- and post-ablation EQ-5D-5L index scores (Table 3). Also, 43 % of patients reported the minimum score in frequency and duration of symptoms (corresponding to “never” for frequency and “not applicable” for duration in combination) in the post-ablation questionnaire. No other multi-item scales in C-CAP1 and C-CAP2 showed problematic ceiling and floor effects.

### Internal consistency

The internal consistency of the items within three disease-specific multi-item scales measured using Cronbach’s alpha was acceptable for both pre- and post-ablation questionnaires (Table 4). Item-total correlation suggested good correlation of each item to the overall score, except in the pre-ablation symptom severity scale whereby “passing out/fainting/blackouts” fitted less well (item-total correlation of 0.135; Table 4). Removal of this item improved the overall Cronbach’s alpha. In the case of both the pre- and post-ablation impact on life scale, removal of the item relating to the financial impact of patient’s palpitations improved overall Cronbach’s alpha (Table 4).

**Table 4:** Measures of internal consistency and test–retest reliability for disease-specific and generic scales within the pre-validation C-CAP1 and C-CAP2 questionnaires

Multi-item scale	Internal consistency measures				Test–retest reliability measures			
	*N*	Cronbach’s alpha	Items with item-total correlation <0.3	Items which if deleted improve overall *α*	*N*	ICC (95 % CIs)	Bland–Altman plot bias value (95 % CIs; *p* value)	Bland–Altman plot limits of agreement
Pre-ablation (C-CAP1)								
Pre-ablation treatment expectations	499	0.705	None	None	82	0.817 (0.730–0.878)	−0.20 (−0.46 to 0.07; *p* = 0.15)	−2.59 to 2.20
Pre-ablation symptom severity	433	0.876	*k* ^a^ (0.135)	*k* ^a^ (*α* if deleted 0.880)	68	0.841 (0.755–0.899)	0.57 (−0.55 to 1.70; *p* = 0.31)	−8.53 to 9.68
Pre-ablation frequency and duration	N/A	N/A	N/A	N/A	86	0.661 (0.523–0.766)	−0.07 (−0.42 to 0.28; *p* = 0.69)	−3.28 to 3.14
Pre-ablation impact on life	483	0.868	None	*i* ^b^ (*α* if deleted 0.872)	77	0.865 (0.796–0.912)	−0.31 (−1.21 to 0.59; *p* = 0.49)	−8.09 to 7.47
Pre-ablation EQ-5D-5L	504	0.772	e^c^ (0.286)	e^c^ (*α* if deleted 0.810)	82	0.845 (0.769–0.897)	−0.02 (−0.05 to 0.00; *p* = 0.09)	−0.27 to 0.22
Pre-ablation EQ-VAS	N/A	N/A	N/A	N/A	87	0.847 (0.775–0.897)	−1.51 (−3.68 to 0.67; *p* = 0.17)	−21.47 to 18.46
Post-ablation (C-CAP2)								
Post-ablation symptom change	412	0.875	None	None	80	0.844 (0.767–0.897)	0.11 (−0.33 to 0.56; *p* = 0.62)	−3.81 to 4.03
Post-ablation symptom severity	377	0.897	None	*k* ^a^ (*α* if deleted 0.899)	65	0.900 (0.840–0.938)	0.77 (−0.15 to 1.69; *p* = 0.10)	−6.53 to 8.07
Post-ablation frequency and duration	N/A	N/A	N/A	N/A	84	0.892 (0.838–0.929)	0.13 (−0.14 to 0.40; *p* = 0.34)	−2.34 to 2.60
Post-ablation impact on life	409	0.918	None	*i* ^b^ (*α* if deleted 0.921)	79	0.946 (0.916–0.965)	0.28 (−0.29 to 0.85; *p* = 0.34)	−4.76 to 5.32
Post-ablation EQ-5D-5L	426	0.830	None	e^c^ (*α* if deleted 0.843)	80	0.807 (0.715–0.872)	−0.01 (−0.04 to 0.02; *p* = 0.50)	−0.28 to 0.26
Post-ablation EQ-VAS	N/A	N/A	N/A	N/A	86	0.929 (0.894–0.953)	−1.28 (−2.94 to 0.37; *p* = 0.13)	−16.40 to 13.83

ICC, intra-class correlation coefficient is a two-way random effects, absolute agreement, single measures model

^a^ *k*: symptom option of “passing out/fainting/blackouts”

^b^ *i*: option relating to “my palpitations have had a financial impact” (e.g. time off work, extra childcare costs)

^c^EQ-5D-5L domain of “anxiety/depression”

### Test–retest reliability

Test–retest reliability was good (ICC ≥ 0.7) for all disease-specific C-CAP scales: patient expectations (C-CAP1 only), symptom change (C-CAP2 only), symptom severity; frequency and duration of symptoms; and impact on life, except the pre-ablation frequency and duration scale (ICC 0.661). C-CAP scales had similar test–retest reliability to EQ-5D-5L and EQ-VAS (Table 4). Of the individual items within the pre-ablation multi-item scales, 23 of 41 had ICCs of ≥0.7. Bland–Altman plots (12) did not indicate bias between test and retest scores, and there was no statistically significant difference between the bias value and 0 (all p values >0.05; Table 4).

The kappa statistic (*κ*) and the proportion of agreement indicated moderate or high agreement between test and retest responses for binary items. The highest was *κ* = 0.969 for “*Is this your first ablation procedure?”* (*n* = 82; *p* < 0.0001; 99 % agreement), and the lowest was *κ* = 0.614 for “*During your hospital stay, or in the month afterwards, did you experience any complications related to your ablation procedure?”* (*n* = 79; *p* < 0.0001; 87 % agreement).

### Construct validity

#### Convergent and divergent validity

Convergent validity was confirmed by the observation of moderate correlations [Spearman’s *ρ* (rho) of between 0.4 and 0.7] for three C-CAP disease-specific multi-item scales from the conditions and symptoms domain shared by C-CAP1 and C-CAP2 (symptom severity; frequency and duration of symptoms; impact on life) against the validated EQ-5D-5L and EQ-VAS tools assumed to be measuring similar concepts. The exception was a mild correlation between EQ-5D-5L/EQ-VAS and pre-ablation “frequency and duration of symptoms” (*ρ* = −0.228 for EQ-5D-5L; *ρ* = −0.221 for EQ-VAS). Correlations between symptom-related multi-item scales within C-CAP were moderate to high (*ρ* ≥ 0.66).

Divergent validity was identified by the observation that disease-related scales in C-CAP1 and C-CAP2 showed low correlation with the “treatment expectation” scale which is measuring a different underlying construct (all were *ρ* < 0.4).

### Responsiveness

Changes from baseline to post-ablation in three disease-specific C-CAP1 and C-CAP2 multi-item scales (symptom severity; frequency and duration of symptoms; impact on life) showed high responsiveness (ES and SRM >0.78; Table 5). Patients reported a 43 % mean improvement in the “symptom severity” scale (a change of −6.6 from a baseline of 15.5; *p* < 0.001), a 45 % improvement in the “symptom frequency and duration” scale (*p* < 0.001), and a 48 % improvement in the “impact on life” scale (*p* < 0.001). General QoL measures showed much smaller ES and SRM values (Table 5). The relative efficiency (RE) value also supports the finding that C-CAP questionnaires are more sensitive than the global measures.

**Table 5:** Pre-ablation and post-ablation scores and effect size measures in disease-specific scales of C-CAP1 and C-CAP2 and generic scales

Multi-item scale	*N*	Mean (SD)			ES	SRM	RE
		Pre-ablation	Post-ablation	Change			
C-CAP disease-specific scales shared across C-CAP1 and C-CAP2							
Symptom severity (score range 0–45)	318	15.5 (8.3)	8.9 (7.9)	−6.6 (8.1) *p* < 0.001	0.80	0.82	6.39
Frequency and duration (score range 0–8)	412	4.7 (2.1)	2.6 (2.6)	−2.1 (2.7) *p* < 0.001	1.03	0.78	7.46
Impact on life (score range 0–30)	372	13.6 (7.4)	7.1 (7.5)	−6.5 (7.4) *p* < 0.001	0.88	0.88	8.59
Generic scales							
EQ-5D-5L (score range −0.594 to 1.00)	416	0.74 (0.22)	0.79 (0.23)	0.05 (0.17)^a^ *p* < 0.001	−0.22	−0.29	1 (Reference)
EQ-VAS (score range 0–100)	426	65.3 (18.8)	72.8 (20.1)	7.4 (17.4)^a^ *p* < 0.001	−0.40	−0.43	2.32

ES, effect size; RE, relative efficiency; SD, standard deviation; SRM, standardised response mean

^a^Increase in EQ-5D-5L and EQ-VAS indicates an improvement in health status

The MCIDs across four anchor questions are shown in Table 5 and demonstrate consistency across the anchors. In the case of the three disease-specific multi-item C-CAP scales (shared across C-CAP1 and C-CAP2), the SEM was considerably smaller than the anchor-based measures of MCID.

The SDC and limits of agreement in the Bland–Altman plot (Table 4) were higher than the anchor-based estimate of MCID for all three disease-specific multi-item C-CAP scales (symptom severity; frequency and duration of symptoms; impact on life; Table 6). As such, on an individual level, the MCID cannot be discriminated from measurement error. Individuals improving beyond the SDC of 9 points on the “symptom severity” scale, 3 points on the “frequency and duration of symptoms scale”, and 8 points on the “impact on life” scale can be interpreted as having undergone a minimal important change. In this sample, participants between 39 and 43 % improved above SDC across the 3 scales.

**Table 6:** Summary of anchor-based and distribution measures of minimal clinically important difference of three multi-item scales shared across C-CAP1 and C-CAP2

	Mean change score in three disease-specific multi-item C-CAP instruments anchored using a range of anchor measures of minimal important change				MCID (mean of anchors)	SEM (pre/post)	SDC (pre/post)
	Symptoms have become less frequent	Symptoms have become shorter	Patient expectations were met	Global health score (EQ-5D-5L) improvement of 20 points			
Symptom severity (score range 0–45)	7.31	6.89	7.22	7.14	7.14	3.29/2.66	9.12/7.38
Frequency and duration (score range 0–8)	1.85	1.45	2.95	0.91	1.79	1.15/0.89	3.19/2.47
Impact on life (score range 0–30)	5.21	5.78	5.93	4.99	5.48	2.80/1.81	7.79/5.02

MCID, minimal clinically important difference; SDC, smallest detectable change; SEM, standard error of measurement

## Discussion

Following incorporation of amendments proposed in this manuscript through the validation process, the final C-CAP questionnaires are valid, reliable, and responsive tools for measuring symptom change in patients undergoing ablation for cardiac arrhythmias (final C-CAP questionnaires are available as an online resource). The final validated C-CAP questionnaires (C-CAP1 and C-CAP2) combine generic global health measures with disease-specific domains to provide a comprehensive picture of the effect that arrhythmias have on patients’ lives. This large validation study builds on previous pilot and content validity work by our research group [1, 8]. The study has demonstrated that C-CAP questionnaires can be used on patients with a range of arrhythmia types and are not limited to those with AF.

The following amendments have been made to produce final version of the C-CAP questionnaires (online resource):

- Removal of *passing out/fainting/blackouts* from the “symptom severity” scale and *my palpitations have had a financial impact* from the “impact on life” scale in C-CAP1 (Q13) and C-CAP2 (Q15)
- Removal of the free text section for medication taken by patients in C-CAP1 (Q15) and C-CAP2 (Q17)
- Amendment of the *N/A* option for “days you have missed at work/school/college” to read “I do not attend work/school/college (✓)”in C-CAP1 (Q9) and C-CAP2 (Q11)
- Removal of the *N/A* option from “days you have had to cut down on your social activities” and “days you have been unable to carry out normal daily activities” questions in C-CAP1 (Q10–11) and C-CAP2 (Q12–13).

We chose to use a classical test theory approach in our psychometric analysis, mainly because of our linear model (pre-/post-testing), our focus on test level scoring, and our relatively small sample size (< 500 subjects) at each measurement point. Further work is being undertaken to compare pre-ablation C-CAP measures with those collected post-ablation, at 1 and 5 years. Incorporation of proposed revisions to the C-CAP questionnaires will be considered for the 5-year follow-up (1-year follow-up uses the pre-validation questionnaire as the validation work was not complete at this study point).

The importance of PROMs as tools to drive improvements in service provision is well recognised [5]. Through future research and use in routine practice, the C-CAP questionnaires provide a tool for UK clinicians and commissioners to collect evidence on whether provision of a cardiac ablation service is having a positive impact on patients which may be difficult to demonstrate through other means. C-CAP has the advantage of enabling comparison of outcomes across different arrhythmia groups, and inclusion of the generic EQ-5D-5L tool allows for wider cross-speciality comparisons [10]. With appropriate further translation and validation work, the C-CAP tool could be used outside of the UK.

The influence of patient expectations on their treatment and recovery has been widely demonstrated [17]. A novel section has been included in C-CAP1 enabling clinicians to explore and manage patient expectations. Appropriate expectation management may improve overall satisfaction with the service. Future analysis will provide an insight into how patient expectations influence the perception of procedural success.

High response rates for C-CAP1 and C-CAP2 indicate that patients find the questionnaires acceptable and that they are not overly burdensome. We identified issues with high numbers of responses to the *not applicable* option for questions of “number of days impacted”. We suspect that some are valid responses but that a proportion may be unreliable. Also the free text format questions relating to medication intake were difficult to validate in any meaningful way. The original purpose was to use a medication dose question to explore changes following ablation; however, a lack of consistency in patients’ responses coupled with the challenges of extrapolating changes in medication regimes as better or worse led us to conclude that this question provided limited value.

Ceiling/floor effects are considered to be an issue if 15 % of patients report maximum and minimum scores [12, 18], and were observed in the “frequency and duration of symptoms”; this may be due in part to patients experiencing a true alleviation of symptoms and also a function of fewer items within the scale. Test–retest reliability was impressive across individual questions and scales and high internal consistency measures were observed. Removal of some items improved the Cronbach’s alpha values.

Disease-specific multi-item C-CAP scales (shared across C-CAP1 and C-CAP2) “symptom severity” and “impact on life” were more responsive to changes following ablation than the global health measures of EQ-5D-5L and EQ-VAS, shown by much larger effect sizes. We have presented distribution-based and anchor-based estimates of MCID alongside SDC values to aid interpretability of quantitative scores. Anchor-based MCIDs are variable because the MCID depends on the definition of “important difference” in the global measure [19]. Several threshold values for SEM have been suggested to estimate MCID; some assert that 1 SEM is roughly equivalent to the minimal important difference determined using anchor-based methods, and others prefer 1.96*SEM [14]. Our results demonstrated that 1.96*SEM came close to the anchor-based method of MCID estimation.

The disadvantage of anchor-based methods is that they do not take into account the measurement precision of the instrument, and alone cannot tell us whether the MCID lies within the measurement error [14]. This study indicated that the SDC is higher than the MCID across three disease-specific C-CAP multi-item scales (symptom severity; frequency and duration of symptoms; impact on life). The study by Lin et al. [20] states that in some instances the MCID scores do not exceed the SDC scores but still convey information about whether a patient group experienced a clinically important change. A 9 point change on the “symptom severity” scale indicates a true and reliable improvement, but a 6–7 point change may be considered clinically meaningful to the patient. The MCID cannot be used to define an important deterioration because we only analysed improved patients and caution should be applied with low baseline scores.

As well as the observation that SDC is higher than the MCID in the disease-specific scales, there were several methodological limitations in this study. Anchor questions were not prospectively designed and included to calculate MCID, although those questions provided adequate proxies for the definition of minimal improvement. Known-groups validity could have been explored in patients for whom their arrhythmia symptoms are adequately controlled by medication. Convergent validity would have been better evaluated by testing another validated AF questionnaire [6] which we would assume measures a similar construct. Future research should test the structure of the C-CAP questionnaires using confirmatory analysis.

The results of this validation study show that the final C-CAP questionnaires (online resource) can be used reliably to measure changes in arrhythmia-related symptom severity, symptom frequency and duration, and impact on life before and after percutaneous cardiac ablation. Additional domains of patient expectations and complications can also be reliably explored using C-CAP1 and C-CAP2. We encourage researchers and clinicians to use C-CAP questionnaires in research and routine clinical settings to measure the impact of ablation services on patients’ QoL (final questionnaires are provided as an Online Resource, copyright Cedar).

## Electronic supplementary material

Below is the link to the electronic supplementary material.

Supplementary material 1 (PDF 158 kb)

Supplementary material 2 (PDF 239 kb)
