# Supplementary material for: Cardiff cardiac ablation patient-reported outcome measure (C-CAP): validation of a new questionnaire set for patients undergoing catheter ablation for cardiac arrhythmias in the UK
Source: Qual Life Res. 2015 Dec 10;25:1571–83. doi: 10.1007/s11136-015-1194-1 (PMC4870294; doi:10.1007/s11136-015-1194-1)
Supplement: Supplementary file 1 — Supplementary material 1 (PDF 158 kb) [file 11136_2015_1194_MOESM1_ESM.pdf]

Supplementary material

**Title:** Cardiff cardiac ablation PROM (C-CAP): a multi-centre validation study of a new questionnaire for patients undergoing catheter ablation for cardiac arrhythmias in the UK

**Journal:** Quality of Life Research

**Authors:** Judith White[1], Kathleen L Withers[1], Mauro Lencioni [2], Grace Carolan-Rees[1], Antony R Wilkes[1], Kathryn A Wood[3], Hannah Patrick[4], David Cunningham[5], Michael Griffith [2]

**Corresponding author:** Judith White (Cedar, Cardiff and Vale University Health Board, Cardiff Medicentre, Heath Park, Cardiff, CF14 4UJ); email: Judith.white3@wales.nhs.uk

# Cardiff Cardiac Ablation PROM (C-CAP1)

Arrhythmia Questionnaire – Before your Operation

Please complete the following and answer **ALL** of the questions as accurately as possible.

**1)** Please tick **ONE** box which best describes what you expect to happen to the **FREQUENCY** of the attacks of your palpitations / fast or irregular heartbeats (i.e. how OFTEN they occur), **after you have recovered from the procedure;**

My palpitations / fast or irregular heartbeats will: (please tick one)

| | |
|---|---|
| Stop | |
| Become less frequent | |
| Will not change | |
| Will become more frequent | |
| I do not have palpitations / fast or irregular heartbeats | |

**2)** Please tick **ONE** box which best describes what you expect to happen to the **LENGTH** of the attacks of your palpitations / fast or irregular heartbeats (i.e. how LONG they last), **after you have recovered from the procedure**;

My palpitations / fast or irregular heartbeats will: (please tick one)

| | |
|---|---|
| Stop | |
| Become shorter | |
| Not change | |
| Become longer | |
| I do not have palpitations / fast or irregular heartbeats | |

**3)** Please tick **ONE** box in **EACH** column which best describes what you expect to happen to your **tiredness and breathlessness** after you have recovered from the procedure**:**

| | Please tick one |
|---|---|
| I will stop feeling tired | |
| I will feel less tired | |
| I will feel no different (tired) | |
| I will feel more tired | |
| I do not feel tired | |

| | Please tick one |
|---|---|
| I will stop feeling breathless | |
| I will feel less breathless | |
| I will feel no different (breathless) | |
| I will feel more breathless | |
| I do not feel breathless | |

**4)** Is this your first ablation procedure? Yes☐ No ☐

**5)** If No, please state how many you have had previously (not including this one) _____

# Cardiff Cardiac Ablation PROM (C-CAP1)

**The following questions are related to your condition and symptoms.**

**6)** Please circle the numbers below that most accurately indicate the severity of each symptom you have had **within the last 30 days**. Please circle **ONE** number for **EVERY** symptom. If you do not have the symptom please circle 0 (None).

| | 0<br>None | 1<br>Mild | 2<br>Moderate | 3<br>Severe |
|---|---|---|---|---|
| Palpitations / fast or irregular heartbeats | 0 | 1 | 2 | 3 |
| Heart flutters | 0 | 1 | 2 | 3 |
| Extra heart beats / missed heart beats | 0 | 1 | 2 | 3 |
| Fatigue / no energy | 0 | 1 | 2 | 3 |
| Dizziness / light-headedness / feeling faint | 0 | 1 | 2 | 3 |
| Hard to catch breath / short of breath | 0 | 1 | 2 | 3 |
| Chest pressure as heart is racing | 0 | 1 | 2 | 3 |
| Headache / migraine | 0 | 1 | 2 | 3 |
| Trouble concentrating | 0 | 1 | 2 | 3 |
| Neck pounding / neck pain / neck discomfort | 0 | 1 | 2 | 3 |
| Trouble sleeping | 0 | 1 | 2 | 3 |
| Tiredness / sleepiness | 0 | 1 | 2 | 3 |
| Nausea / vomiting | 0 | 1 | 2 | 3 |
| Anxiety / fear / worry | 0 | 1 | 2 | 3 |

**7)** How often do you usually get palpitations / fast or irregular heartbeats? Please tick one only

| Never | Once a month or less | Several times a month | Several times a week | Several times a day |
|---|---|---|---|---|
| ☐ | ☐ | ☐ | ☐ | ☐ |

**8)** How long do your episodes of palpitations / fast or irregular heartbeats **usually** last? Please tick one only.

| Not Applicable | Less than 5 minutes | 5 minutes to 1 hour | More than 1 hour but less than 12 hours | 12 hours or more |
|---|---|---|---|---|
| ☐ | ☐ | ☐ | ☐ | ☐ |

**9)** Have your palpitations / fast or irregular heartbeats had any impact on the number of days you have attended work / school / college (including unpaid work, role as a carer and time spent job-seeking) **in the last 30 days**? If so, for how many days do you think it had an impact?

| | No of Days (0-30) | I do not attend work/ school/ college (✓) |
|---|---|---|
| Days you have missed at work / school / college | | |

**10)** Have your palpitations / fast or irregular heartbeats had any impact on your social activities **in the last 30 days**, and if so, for how many days do you think it has had an impact?

| | No of Days (0-30) |
|---|---|
| Days you have had to cut down on your social activities | |

**11)** Have your palpitations / fast or irregular heartbeats had any impact on the number of days you have been able to carry out your normal daily activities (including household duties) **in the last 30 days**? If so, for how many days do you think it has had an impact?

| | No of Days (0-30) |
|---|---|
| Days you have been unable to carry out normal daily activities | |

**12)** How many times have you needed to visit a GP / Hospital **within the last 30 days** (related to your palpitations / fast or irregular heartbeats)?

GP ☐ Hospital ☐

**13)** Please circle the number that most accurately indicates how you feel about the following statements. Please circle **ONE** number for **EVERY** statement. If you feel the statement does not apply to you please circle 0 (Not Applicable).

| | 0 Not Applicable | 1 Agree Mildly | 2 Agree Moderately | 3 Agree Strongly |
|---|---|---|---|---|
| I worry that my palpitations/fast or irregular heartbeats will start | 0 | 1 | 2 | 3 |
| My everyday physical activities are limited | 0 | 1 | 2 | 3 |
| My palpitations have an impact on my own sport / leisure activities | 0 | 1 | 2 | 3 |
| I worry about the effect of my heart rhythm on my health | 0 | 1 | 2 | 3 |
| My palpitations / fast or irregular heartbeats interfere with my social activities | 0 | 1 | 2 | 3 |
| I am restricted in my travel / holiday plans | 0 | 1 | 2 | 3 |
| I am less confident due to my palpitations | 0 | 1 | 2 | 3 |
| My palpitations / fast or irregular heartbeats have an emotional / physical impact when I am driving | 0 | 1 | 2 | 3 |
| My palpitations have an impact on my family / friends | 0 | 1 | 2 | 3 |

**14)** Do you normally take any medication for your palpitations / fast or irregular heartbeat?

Yes ☐ No ☐

**15)** If you currently take medication for your palpitations / fast or irregular heartbeats how desirable is it for you to reduce or stop taking this medication following your ablation?

| Not applicable | Not important | Quite important | Very important |
|---|---|---|---|
| ☐ | ☐ | ☐ | ☐ |

**The following questions are related to your GENERAL health:**

**16)** Have you been told by a doctor that you have any of the following? (Please tick all that apply)

| | | | |
|---|---|---|---|
| Liver disease | ☐ | High blood pressure | ☐ |
| Other heart condition e.g. angina, heart attack or heart failure | ☐ | Cancer (within the last 5 years) | ☐ |
| Diabetes | ☐ | Leg pain when walking due to poor circulation | ☐ |
| Lung disease e.g. asthma, chronic bronchitis or emphysema | ☐ | Arthritis | ☐ |
| Kidney disease | ☐ | Depression | ☐ |
| Problems caused by stroke | ☐ | Diseases of the nervous system e.g. Parkinson's disease or multiple sclerosis | ☐ |

Cardiff Cardiac Ablation PROM (C-CAP) pre-ablation questionnaire by Cedar is licensed under a Creative Commons Attribution-ShareAlike 4.0 International License. (cc) BY-SA
