# Supplementary material for: Cardiff cardiac ablation patient-reported outcome measure (C-CAP): validation of a new questionnaire set for patients undergoing catheter ablation for cardiac arrhythmias in the UK
Source: Qual Life Res. 2015 Dec 10;25:1571–83. doi: 10.1007/s11136-015-1194-1 (PMC4870294; doi:10.1007/s11136-015-1194-1)
Supplement: Supplementary file 2 — Supplementary material 2 (PDF 239 kb) [file 11136_2015_1194_MOESM2_ESM.pdf]

Supplementary material

Title: Cardiff cardiac ablation PROM (C-CAP): a multi-centre validation study of a new questionnaire for patients undergoing catheter ablation for cardiac arrhythmias in the UK

Journal: Quality of Life Research

Authors: Judith White[1], Kathleen L Withers[1], Mauro Lencioni [2], Grace Carolan-Rees[1], Antony R Wilkes[1], Kathryn A Wood[3], Hannah Patrick[4], David Cunningham[5], Michael Griffith [2]

Corresponding author: Judith White (Cedar, Cardiff and Vale University Health Board, Cardiff Medicentre, Heath Park, Cardiff, CF14 4UJ); email: Judith.white3@wales.nhs.uk

Cardiff Cardiac Ablation PROM (C-CAP2)

Arrhythmia Questionnaire – After your Operation

We would be grateful if you could give us some feedback on how you have been feeling since your procedure to treat your palpitations / fast or irregular heartbeats. Please answer ALL of the questions.

1) Please tick **ONE** box which best describes your symptoms **within the last 30 days** related to the **FREQUENCY** of the attacks of your palpitations / fast or irregular heartbeats (i.e. how OFTEN they occur):

My palpitations / fast or irregular heartbeats have: (please tick one)

Stopped	
Become less frequent	
Not changed	
Become more frequent	

2) Please tick **ONE** box which best describes your symptoms **within the last 30 days** related to the **LENGTH** of the attacks of your palpitations / fast or irregular heartbeats (i.e. how LONG they last):

The duration of my palpitations / fast or irregular heartbeats has: (please tick one)

They have stopped	
Become shorter	
Not changed	
Become longer	

3) Please tick **ONE** box in **EACH** column which best describes your symptoms **within the last 30 days** related to your **tiredness and breathlessness**:

	Please tick one
I do not feel tired	
I feel less tired	
I feel no different (tiredness)	
I feel more tired	

	Please tick one
I do not feel breathless	
I feel less breathless	
I feel no different (breathlessness)	
I feel more breathless	

4) During your hospital stay, or in the month afterwards, did you experience any complications related to your ablation procedure?

Yes ☐ No ☐

5) Which complications (if any) were you **WARNED ABOUT** related to your procedure? Please tick ALL that you were warned about.

None	
Excessive bruising	
Wound site complications (eg. bleeding / swelling)	
Stroke	
Blood around the heart requiring a needle to remove it	
Air around the lung (pneumothorax)	
Extended hospital stay (related to your procedure)	
Readmission to hospital (related to your procedure)	

6) Which complications (if any) did you **EXPERIENCE** related to your procedure? Please tick ALL that you experienced:

No complications	
Excessive bruising	
Wound site complications (e.g. bleeding / swelling)	
Stroke	
Blood around the heart requiring a needle to remove it	
Air around the lung (pneumothorax)	
Extended hospital stay (related to your procedure)	
Readmission to hospital (related to your procedure)	
Other complication related to your procedure (please specify)	

7) Did the outcome of the procedure meet or exceed your expectations?

Yes ☐ No ☐

The following questions are related to your condition and symptoms SINCE your recent procedure

8) Please circle the numbers below that most accurately indicate the severity of each symptom you have had **within the last 30 days**. Please circle **ONE** number for **EVERY** symptom. If you do not have the symptom please circle 0 (None).

	0 None	1 Mild	2 Moderate	3 Severe
Palpitations / fast or irregular heartbeats	0	1	2	3
Heart flutters	0	1	2	3
Extra heart beats / missed heart beats	0	1	2	3
Fatigue / no energy	0	1	2	3
Dizziness / light-headedness / feeling faint	0	1	2	3
Hard to catch breath / short of breath	0	1	2	3
Chest pressure as heart is racing	0	1	2	3
Headache / migraine	0	1	2	3
Trouble concentrating	0	1	2	3
Neck pounding / neck pain / neck discomfort	0	1	2	3
Trouble sleeping	0	1	2	3
Tiredness / sleepiness	0	1	2	3
Nausea / vomiting	0	1	2	3
Anxiety / fear / worry	0	1	2	3

9) Since your ablation, how often do you usually get palpitations / fast or irregular heartbeats? **Please tick one only**

Never	Once a month or less	Several times a month	Several times a week	Several times a day
☐	☐	☐	☐	☐

10) Since your ablation, how long do your episodes of palpitations / fast or irregular heartbeats **usually** last? **Please tick one only**

Not Applicable	Less than 5 minutes	5 minutes to 1 hour	More than 1 hour but less than 12 hours	12 hours or more
☐	☐	☐	☐	☐

11) Have your palpitations / fast or irregular heartbeats had any impact on the number of days you have attended work / school / college (including unpaid work, role as a carer and time spent job-seeking) **in the last 30 days**? If so, for how many days do you think it had an impact?

	No of Days (0-30)	I do not attend work/ school/ college (✓)
Days you have missed at work / school / college		

12) Have your palpitations / fast or irregular heartbeats had any impact on your social activities **in the last 30 days**, and if so, for how many days do you think it has had an impact?

	No of Days (0-30)
Days you have had to cut down on your social activities	

13) Have your palpitations / fast or irregular heartbeats had any impact on the number of days you have been able to carry out your normal daily activities (including household duties) **in the last 30 days**? If so, for how many days do you think it has had an impact?

	No of Days (0-30)
Days you have been unable to carry out normal daily activities	

14) How many times have you needed to visit a GP / Hospital **within the last 30 days** (related to your palpitations / fast or irregular heartbeats)?

GP ☐ Hospital ☐

15) Please circle the number that most accurately indicates how you feel about the following statements, please circle **ONE** number for **EVERY** statement. If you feel the statement does not apply to you please circle 0 (Not Applicable).

	0 Not Applicable	1 Agree Mildly	2 Agree Moderately	3 Agree Strongly
I worry that my palpitations/fast or irregular heartbeats will start	0	1	2	3
My everyday physical activities are limited	0	1	2	3
My palpitations have an impact on my own sport / leisure activities	0	1	2	3
I worry about the effect of my heart rhythm on my health	0	1	2	3
My palpitations / fast or irregular heartbeats interfere with my social activities	0	1	2	3
I am restricted in my travel / holiday plans	0	1	2	3
I am less confident due to my palpitations	0	1	2	3
My palpitations / fast or irregular heartbeats have an emotional / physical impact when I am driving	0	1	2	3
My palpitations have an impact on my family / friends	0	1	2	3

16) Do you normally take any medication for your palpitations / fast or irregular heartbeat?

Yes ☐ No ☐

17) If you take medication for your palpitations / fast or irregular heartbeats is it?

a) More than before the procedure ☐
b) The same as before the procedure ☐
c) Less than before the procedure ☐

The following questions are related to your GENERAL health:

18) Have you been told by a doctor that you have any of the following?
(Please tick all that apply)

Liver disease	☐	High blood pressure	☐
Other heart condition e.g. angina, heart attack or heart failure	☐	Cancer (within the last 5 years)	☐
Diabetes	☐	Leg pain when walking due to poor circulation	☐
Lung disease e.g. asthma, chronic bronchitis or emphysema	☐	Arthritis	☐
Kidney disease	☐	Depression	☐
Problems caused by stroke	☐	Diseases of the nervous system e.g. Parkinson's disease or multiple sclerosis	☐

Cardiff Cardiac Ablation PROM (C-CAP) post ablation questionnaire by Cedar is licensed under a Creative Commons Attribution-ShareAlike 4.0 International License. (cc) BY-SA
